# Chronic Respiratory Aeroallergen Exposure in Mice Induces Epithelial-Mesenchymal Transition in the Large Airways

**DOI:** 10.1371/journal.pone.0016175

**Published:** 2011-01-20

**Authors:** Jill R. Johnson, Abraham Roos, Tove Berg, Magnus Nord, Jonas Fuxe

**Affiliations:** 1 Division of Vascular Biology, Department of Medical Biochemistry and Biophysics, Karolinska Institutet, Stockholm, Sweden; 2 Lung Research Laboratory, Division for Respiratory Medicine, Department of Medicine, Karolinska Institutet, Karolinska University Hospital-Solna, Stockholm, Sweden; Stockholm University, Sweden

## Abstract

Chronic allergic asthma is characterized by Th2-polarized inflammation and leads to airway remodeling and fibrosis but the mechanisms involved are not clear. To determine whether epithelial-mesenchymal transition contributes to airway remodeling in asthma, we induced allergic airway inflammation in mice by intranasal administration of house dust mite (HDM) extract for up to 15 consecutive weeks. We report that respiratory exposure to HDM led to significant airway inflammation and thickening of the smooth muscle layer in the wall of the large airways. Transforming growth factor beta-1 (TGF-β1) levels increased in mouse airways while epithelial cells lost expression of E-cadherin and occludin and gained expression of the mesenchymal proteins vimentin, alpha-smooth muscle actin (α-SMA) and pro-collagen I. We also observed increased expression and nuclear translocation of Snail1, a transcriptional repressor of E-cadherin and a potent inducer of EMT, in the airway epithelial cells of HDM-exposed mice. Furthermore, fate-mapping studies revealed migration of airway epithelial cells into the sub-epithelial regions of the airway wall. These results show the contribution of EMT to airway remodeling in chronic asthma-like inflammation and suggest that Th2-polarized airway inflammation can trigger invasion of epithelial cells into the subepithelial regions of the airway wall where they contribute to fibrosis, demonstrating a previously unknown plasticity of the airway epithelium in allergic airway disease.

## Introduction

Allergic asthma is caused by respiratory exposure to common aeroallergens like house dust mite (HDM) and results in reversible airway obstruction, chronic Th2-polarized inflammation and damage to the airway epithelium [1], [2]. These events have been associated with a dysregulated repair process, which is characterized by elevated expression of TGF-β and EGF and ultimately results in airway fibrosis and lung dysfunction [3]. Epithelial-to-mesenchymal transition (EMT) is an important mechanism during development and cancer progression whereby epithelial cells gain the capacity to migrate out of their context through down-regulation of epithelial markers, such as junction proteins and cytokeratins, and gained expression of mesenchymal proteins, such as vimentin and α-SMA [4], [5]. EMT also results in the acquisition of stem cell features, linking EMT to the generation of cancer stem cells [6], [7]. Transforming growth factor-beta (TGF-β) is a major inducer of EMT [4], [8] and is secreted by various cells including infiltrating immune cells [9], [10]. TGF-β-induced EMT is driven by transcription factors including Smad, Snail, Zeb, Twist and AP-1, which form complexes that either repress epithelial genes or activate mesenchymal genes [6], [8], [11], [12]. TGF-β has also been shown to synergize with EGF to induce EMT in various cell types [13], [14].

It has been postulated that EMT can be triggered under inflammatory conditions and contribute to cancer metastasis and organ fibrosis [4], [5], [15], [16]. Th2 lymphocytes can enhance the spread of tumor cells to distal sites via the activation of TGF-β- and EGF-expressing tumor-associated macrophages [3], [17] suggesting that a Th2-polarized immune response may promote tumor cell dissemination [18]. Previous *in vitro* studies have demonstrated that HDM proteins can cooperate with TGF-β and EGF to promote EMT in cultured airway epithelial cells by stimulating internalization of E-cadherin [19], cleavage of junction proteins [20] and activation of the protease-activated receptor PAR-2 [21]. However, it is currently not known whether EMT contributes to airway remodeling in asthma and whether chronic allergic inflammation is sufficient to trigger this process.

In this study, we asked if EMT could contribute to airway remodeling in a chronic Th2-polarized inflammatory microenvironment driven by respiratory aeroallergen exposure. We evaluated this process by employing airway epithelial cell-fate tracking in mice with chronic allergic asthma induced by exposure to house dust mite extract (HDM), a common environmental aeroallergen. We have identified EMT as a significant contributor to airway wall thickening in severe asthma and confirmed the role of TGF-β and EGF signaling in dysregulated repair processes in the lung.

## Methods

### Animals

Reporter mice were constructed by crossing Rosa26stop-LacZ reporter mice (B6;129S4-Gt(ROSA)26Sortm1Sor/J; Jackson Laboratoies) with mice expressing Cre under the surfactant protein C (SPC) promoter (SPC-Cre, generously provided by Brigid Hogan at Duke University Medical Center) to generate transgenic mice stably expressing LacZ in lung epithelial cells (SPC-Cre;R26stop-LacZ). Male and female mice were bred in-house at the Karolinska Institutet animal facility at the Department of Mikrobiologi, Tumör- och Cellbiologi (MTC) and initiated into experiments at 8–12 weeks of age. Mice were housed under specific pathogen-free conditions following a 12-h light-dark cycle and were provided food and water ad libitum. Mice were exposed to purified HDM whole-body extract (Greer Laboratories, Lenoir, North Carolina, USA) intranasally (25 µg protein in 10 µL saline) under inhaled anesthesia (Isoflurane; Baxter Chemical, Kista, Sweden) for five consecutive days, followed by two days rest, for five, ten or fifteen consecutive weeks. Negative control animals were administered 10 µL saline intranasally daily on the same schedule. No exogenous adjuvant was given at any time. All experiments described in this study were approved by the Research Ethics Committee at the Karolinska Institute.

### Collection and measurement of specimens

72 hours after the last allergen administration, mice were anesthetized with fluanison/fentanyl/midazolam (0.1 mL/10 g body weight i.p.), followed by full-body perfusion with PBS via the left cardiac ventricle. Bronchoalveolar lavage fluid (BAL) was collected by dissection of the lungs and cannulation of the trachea with polyethylene tubing (Becton Dickinson, Sparks, MA). Briefly, the lungs were lavaged twice with PBS (0.25 mL followed by 0.2 mL). Approximately 0.3 mL of the instilled fluid was consistently recovered. Total cell counts in BAL fluid were performed using a Countess automated cell counter (Invitrogen). After centrifugation, cell pellets were resuspended in PBS and smears were prepared by cytocentrifugation (Sakura Finetek Europe BV, Zoeterwoude, the Netherlands) at 300 rpm for 2 minutes. Smears were fixed, stained with hematoxylin and eosin and differential counts of BAL cells were determined from at least 300 leukocytes using standard hematological criteria to classify them as mononuclear cells, neutrophils or eosinophils.

### Cell Culture

A549 cells (ATCC, Manassas, VA) and 16HBE14o- cells (kindly provided by Dr. D.C. Gruenert, University of California, San Francisco) were cultivated in Ham's F12 medium or Minimum Essential Medium (MEM; Invitrogen, Stockholm, Sweden), respectively, supplemented with 1% penicillin/streptomycin, 1% L-glutamine and 10% fetal bovine serum (FCS; Invitrogen). Prior to the addition of growth factors, cells were transferred into low serum medium (1% FCS) and subsequently treated with 10 ng/mL TGF-β (R&D Systems, Abingdon, UK) and/or 50 ng/mL EGF (R&D Systems) for 72 h. Cells were then fixed for immunofluorescent staining or lysed for RNA analysis.

### Immunofluorescent staining of cultured cells

Cells which had been grown on coverslips in a 12-well plate were fixed in absolute ethanol for 15 min at RT (for the detection of junction proteins) or 3% PFA for 20 min at RT (for the detection of transcription factors), washed three times with PBS and quenched with 10 mM glycine for 20 min at RT. Following two additional PBS washes, cells to be stained for Snail1/pSmad3 were permeabilized with 0.1% Triton-X in PBS for 30 min at RT, washed twice with PBS, then treated with 8 M urea in PBS for 3 min at RT. Following two more PBS washes, cells were incubated in blocking buffer (PBS with 0.1% BSA and 5% normal goat serum) for 30 min RT, then incubated with primary antibodies diluted in PBS with 0.1% BSA for 60 min RT. The primary antibodies used in this analysis were: rabbit anti-CAR (1∶500), mouse anti-E-cadherin (1∶500; clone 36, BD Biosciences), mouse anti-vimentin (1∶50, 3B4, DAKO), rat anti-occludin (1∶3, clone MOC37), mouse anti-SNAIL1 (1∶3) and rabbit anti-phosphorylated Smad3 (1∶100; #9520, Cell Signaling Technology, Danvers, MA). Cells were washed twice with PBS with 0.1% BSA, then incubated with secondary antibodies for 30 min at RT. The species-specific fluorescently-labeled secondary antibodies used in this study were: DyLight™ 488-conjugated goat anti-rabbit IgG, DyLight™ 488-conjugated goat anti-rat IgG and DyLight™ 549-conjugated goat anti-mouse IgG; all used 1∶500 from Jackson ImmunoResearch Europe Ltd., Newmarket, UK. Following two washes in PBS with 0.1% BSA and two final washes with PBS, coverslips were removed from the wells, mounted and imaged on a Nikon Eclipse E800 microscope.

### Semi-quantitative real-time PCR

Total RNA was isolated from A549 and 16HBE14o- cells and purified using the RNeasy Mini Kit (Qiagen, Stockholm, Sweden), supplemented with the RNase-Free DNase Set (Qiagen). RNA from blood was purified using the the mouse RiboPure™-blood RNA Isolation Kit (Ambion). cDNA was obtained using the iScript Select cDNA Synthesis Kit (Bio-Rad Laboratories AB, Stockholm, Sweden), and the absence of DNA contamination was verified by excluding the reverse transcriptase from subsequent PCR reactions. cDNA aliquots were subjected to PCR using the QuantiTect SYBR Green PCR Kit (Qiagen) to amplify human CAR, occludin, E-cadherin, vimentin, SNAIL1, α-smooth muscle actin (α-SMA) and GAPDH with primers using QuantiTect Primer Assays (Qiagen). Each PCR reaction was carried out as follows: 15 min at 95°C, 15 sec at 94°C, 30 sec at 55°C, and 30 sec at 72°C. Each cycle was repeated 35 times following the manufacturer's recommendations using a Rotorgene RG-3000A thermal cycler, and Rotorgene 6.0 software (Corbett Research, Umeå, Sweden). Based on the comparative Ct method, gene expression levels were calculated and GAPDH was used as a control gene. Untreated control samples for each cell line were set to 100% and fold change in expression in following treatment are represented as bar graphs ± standard error of the mean.

### Histology

Lungs were inflated with 1% paraformaldehyde via the trachea and stored in 1% paraformaldehyde for an additional 30 minutes before being transferred to PBS. The lungs were dissected away from the trachea, cryopreserved in 30% sucrose overnight at 4°C, embedded in OCT (Sakura) and cut at a thickness of 5 µm (for hematoxylin and eosin staining) or 15 µm (for immunofluorescent imaging). For hematoxylin and eosin staining, sections were fixed in 100% methanol and stained according to a standard protocol. Forr immunofluorescent staining, sections were blocked with 5% normal goat serum in PBS containing 0.1% BSA and 0.3% Triton-X for 1 hour at RT, then stained with the following primary antibodies: chicken anti-β-galactosidase (1∶500; ab9361, Abcam, Cambridge, UK), rabbit anti-vimentin (1∶500; ab45939, Abcam), rat anti-E-cadherin (1∶250; ab11512, Abcam), rabbit anti-occludin (1∶500; 71–1500, Zymed/Invitrogen), rabbit anti-procollagen I (1∶500; generously provided by K. Tryggvason, Karolinska Institutet, Stockholm, Sweden), rat anti-Snail1 (1∶500; generously provided by K.-F. Becker, Technische Universität München, Munich, Germany), rabbit anti-phosphorylated Smad3 (1∶100; #9520, Cell Signaling Technology, Danvers, MA) and CY3-conjugated mouse anti-α-SMA (1∶1000; clone 1A4, Sigma-Aldrich, St. Louis, MO).

The species-specific fluorescently-labeled secondary antibodies used in this study were: DyLight™ 488-conjugated goat anti-chicken IgY, DyLight™ 549-conjugated goat anti-rabbit IgG, DyLight™ 549-conjugated goat anti-rat IgG, DyLight™ 649-conjugated goat anti-rabbit IgG and DyLight™ 649-conjugated goat anti-rat IgG; all used 1∶500 from Jackson ImmunoResearch Europe Ltd., Newmarket, UK. Isotype control staining for Snail/pSmad3 was performed using rat IgG2a (BD Biosciences) labeled with DyLight™ 549-conjugated goat anti-rat IgG and an affinity-purified polyclonal rabbit IgG labeled with DyLight™ 649-conjugated goat anti-rabbit IgG. Negative control images (with omission of the primary antibody) and istotype control images for lung sections taken from either saline control mice or from mice exposed to HDM for 15 weeks (as indicated) are shown in Fig. S1. Tissues were imaged using a Zeiss LSM510 confocal microscope and evaluated using LSM510 software.

### Data analysis

Data were analyzed using GraphPad InStat (GraphPad Software, Inc., La Jolla, CA), and are expressed as mean ± SEM, unless otherwise indicated. Results were interpreted using ANOVA followed by the Tukey post hoc test (where applicable). Differences were considered to be statistically significant when p<0.05.

## Results

### Generation of transgenic mice with permanently labeled airway epithelial cells

**Figure 1:**
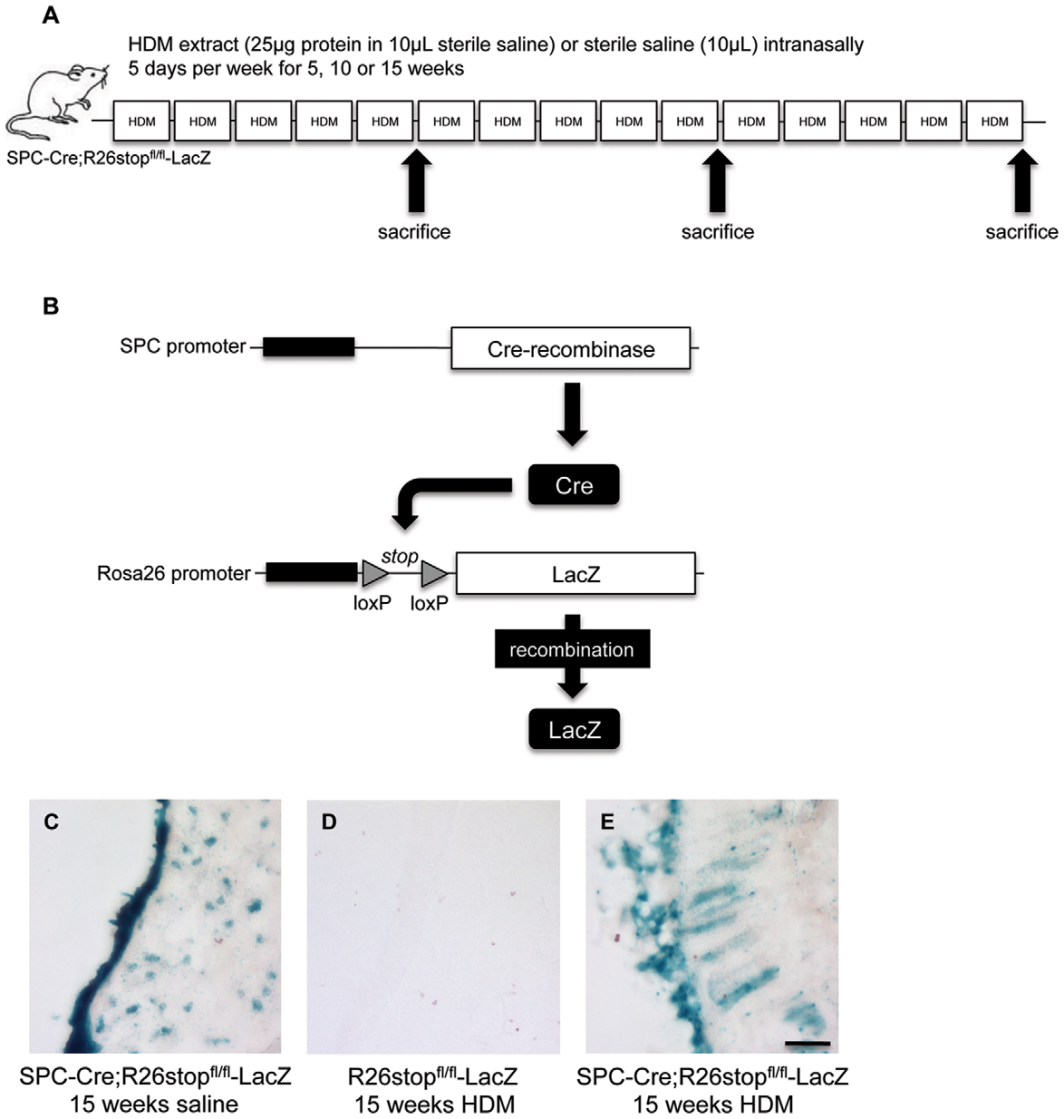
LacZ expression pattern in SPC-Cre;R26stop^fl/fl^-LacZ mice. (A) Model of chronic respiratory house dust mite (HDM) extract exposure. Mice were administered sterile saline or 25 µg of HDM extract in a volume of 10 µL 5 days a week for up to 15 consecutive weeks. Mice were sacrificed after 5, 10 or 15 weeks of HDM exposure. (B) Characterization of the reporter mice used in this study. Cre is expressed by the SPC promoter and removes the floxed stop sequence in front of the gene for LacZ under the control of the ROSA26 promoter. Thus, all lung epithelial cells stably and irreversibly express LacZ. (C–E) Enzymatic staining for β-galactosidase activity was performed on 15 µm-thick lung sections from (C) SPC-Cre;R26stop^fl/fl^-LacZ mice exposed to saline, (D) R26stop^fl/fl^-LacZ mice exposed to HDM and (E) SPC-Cre;R26stop^fl/fl^-LacZ exposed to HDM. Scale bar 10 µm.

To determine whether chronic allergic airway inflammation caused by respiratory exposure to HDM extract (**Fig. 1A**) results in EMT *in vivo*, we employed an established transgenic mouse model in which mice expressing a LacZ reporter gene under the Rosa26 promoter (B6;129S4-Gt(ROSA)26Sortm1Sor/J) were crossed with mice expressing Cre recombinase by the human surfactant protein C promoter (SPC-Cre), which is restrictively active in bronchiolar and alveolar epithelial cells (**Fig. 1B**) [22]. This resulted in the generation of double transgenic mice (SPC-Cre;R26stop^fl/fl^-LacZ), in which airway epithelial cells were permanently labeled with LacZ (**Fig. 1C**), allowing us to track the acquisition of mesenchymal markers in these cells in the context of chronic allergic airway inflammation.

### Induction of EMT in HDM-induced chronic airway inflammation

**Figure 2:**
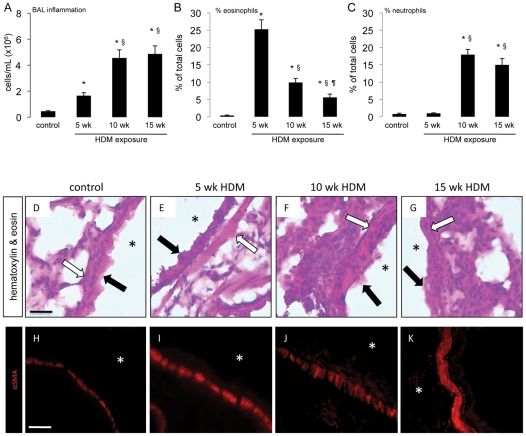
Prolonged respiratory HDM exposure induces inflammation. Mice were administered sterile saline or 25 µg of HDM extract in a volume of 10 µL 5 days a week for 5, 10 or 15 consecutive weeks. (A–C) Bronchoalveolar lavage (BAL) analysis was performed to determine total inflammatory cell infiltrate (A) and to differentiate between eosinophils (B) and neutrophils (C). * *p*<0.05 compared to saline control animals, § *p*<0.05 compared to mice exposed to HDM for 5 weeks and ¶ *p*<0.05 compared to mice exposed to HDM for 10 weeks. Data represent mean ± SEM, n = 10–15 mice per group from two independent experiments. (D–G) Chemical staining for hematoxylin and eosin was performed on 5 µm-thick lung sections from (D) control mice exposed to saline, (E) mice exposed to HDM for 5 weeks, (F) 10 weeks or (G) 15 weeks. * indicate airway lumen, closed arrows indicate the epithelium, open arrows indicate airway smooth muscle. (H–K) Immunofluorescent staining for α-smooth muscle actin (α-SMA) was performed on 15 µm-thick lung sections from (H) control mice exposed to saline, (I) mice exposed to HDM for 5 weeks, (J) 10 weeks or (K) 15 weeks. * indicate airway lumen. Scale bar 10 µm.

Exposure to HDM extract lead to robust airway inflammation (**Fig. 2A**), characterized by eosinophil infiltration after 5 weeks (**Fig. 2B**) and neutrophil infiltration after 10 and 15 weeks of allergen exposure (**Fig. 2C**), as previously described [23]. Histological analysis of lung tissue from these mice demonstrated significant inflammation and epithelial damage associated with HDM exposure (**Fig. 2D–G**). We also observed marked thickening of the sub-epithelial contractile smooth muscle layer tissue in HDM-exposed mice (**Fig. 2H–K**), a feature of chronic allergic asthma [24] which can only be partially accounted for by smooth muscle hyperplasia and infiltration of bone-marrow derived myofibroblasts [25].

**Figure 3:**
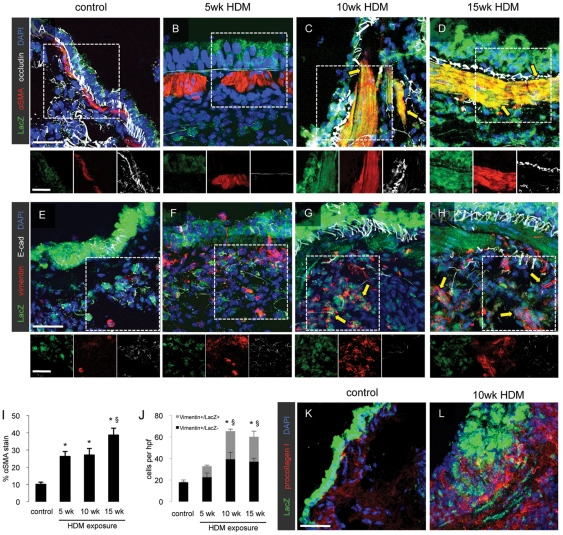
Prolonged respiratory HDM exposure induces epithelial-to-mesenchymal transition. Lung sections (15 µm thick) were prepared from control mice and mice exposed to HDM for 5, 10 or 15 weeks and immunofluorescent staining for the co-expression of the LacZ reporter in airway epithelial cells with α-SMA and occludin (A–D) and with vimentin and E-cadherin (E–H) was performed. Scale bars 10 µm. Quantification of lung fibrosis was performed by morphometric analysis of lung sections stained for α-SMA (I) or LacZ and vimentin (J). * *p*<0.05 compared to saline control animals, § *p*<0.05 compared to mice exposed to HDM for 5 weeks. Data represent mean ± SEM, n = 5 mice per group. Additional lung sections were stained to detect procollagen I-producing cells in the airway wall in control mice and in mice exposed to HDM for 10 weeks (K, L).

Immunofluorescent staining of lung sections revealed decreased staining of occludin and E-cadherin and increased expression of vimentin in the airway epithelium after 5 weeks of HDM exposure (**Fig. 3B, F**) compared to control (**Fig. 3A, E**). These changes were accentuated after 10 weeks and 15 weeks of HDM exposure (**Fig. 3C, D, G, H**). Airway smooth muscle mass as determined by immunostaining for α-SMA was significantly elevated at all time points after HDM exposure (**Fig. 3I**). After 10 and 15 weeks of HDM exposure, LacZ+/α-SMA+ cells were incorporated into airway smooth muscle (arrows, **Fig. 3C, D**) and LacZ+/vimentin+ cells were present in the sub-epithelium (arrows, **Fig. 3G, H**). Quantification of LacZ+/vimentin+ cells demonstrated that approximately one third of the vimentin-positive cell population was positive for LacZ (**Fig. 3J**). Additionally, expression of pro-collagen I was detected in the airway epithelial cells of mice following 10 weeks of HDM challenge (**Fig. 3K, L**).

### Chronic HDM exposure leads to TGF-β expression and activation of TGF-β signaling pathways in the lung epithelium

**Figure 4:**
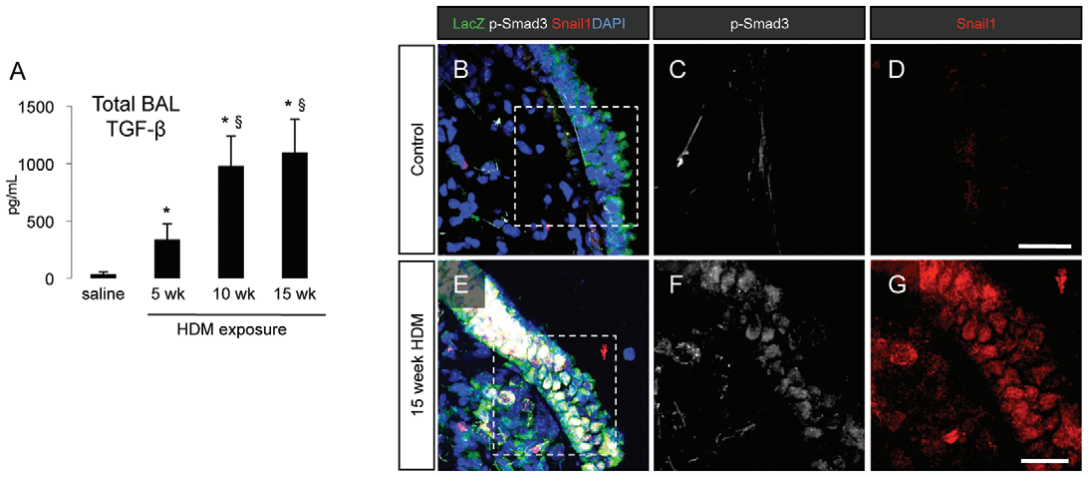
Activation of TGF-β signaling pathways following chronic HDM exposure. (A) Analysis of TGF-β levels in mouse bronchoalveolar lavage (BAL) fluid in saline controls and mice exposed to HDM for 5, 10 or 15 weeks. BAL fluid was collected at the time of sacrifice and analyzed by ELISA for the expression of mouse TGF-β1. * *p*<0.05 compared to saline control animals, § *p*<0.05 compared to mice exposed to HDM for 5 weeks. Data represent mean ± SEM, n = 8 per group from two independent experiments. (B–G) Immunofluorescent staining for the expression of LacZ, p-Smad3 and Snail1 in lung sections from control mice and mice exposed to HDM for 15 weeks.

Bronchoalveolar lavage (BAL) analysis demonstrated a significant and progressive increase in TGF-β1 protein levels in the lavage fluid from mouse airways with sustained exposure to HDM (**Fig. 4** **A**). Activation of Smad-dependent TGF-β signaling pathways was demonstrated by immunofluorescent localization of phosphorylated Smad3 (p-Smad3) and Snail1 in the nuclei of airway epithelial cells after 15 weeks of HDM exposure (**Fig. 4E–G**) but not under baseline conditions (**Fig. 4B–D**).

### Cooperative induction of EMT in airway epithelial cells by TGF-β and EGF

**Figure 5:**
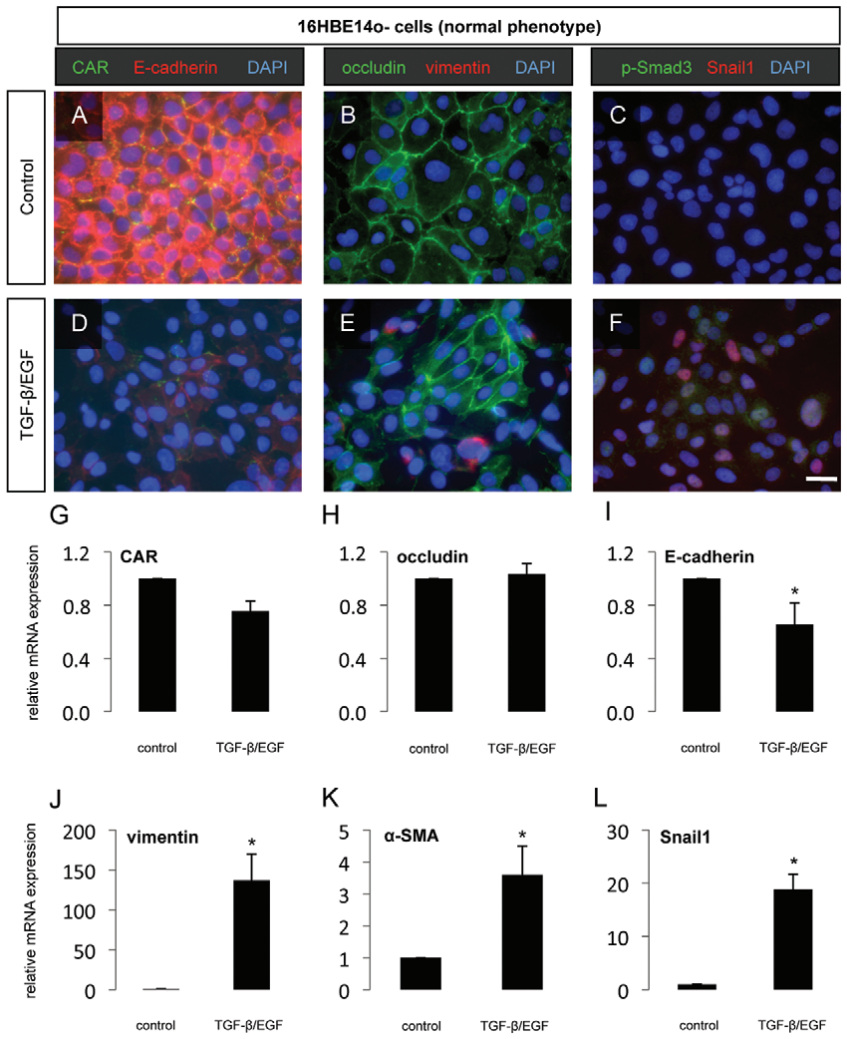
Partial induction of EMT in 16HBE14o- cells. (A–I) Progression through EMT was evaluated in the phenotypically normal human lung epithelial cell line 16HBE14o- 72 h after the addition of TGF-β1 (10 ng/mL) and EGF (50 ng/mL) to the culture medium. The epithelial junction proteins CAR and E-cadherin (A, D), the tight junction protein occludin and the mesenchymal marker vimentin (B, E) as well as the EMT-associated transcription factors pSmad3 and Snail1 (C, F) were assessed by immunofluorescent staining. Scale bar 10 µm. Quantification of the relative mRNA expression of CAR (G), occludin (H), E-cadherin (I), vimentin (J), α-SMA (K) and Snail1 (L) was assessed by qPCR. * *p*<0.05 compared to untreated cells. Data represent mean ± SEM, n = 6 from two independent experiments.

Immunofluorescent analysis of 16HBE14o- cells, which usually express high levels of the epithelial tight junction proteins coxsackie and adenovirus receptor (CAR) and occludin and the adherens junction protein E-cadherin, showed dramatically reduced expression of these epithelial proteins after 72 h of treatment with TGF-β/EGF (Fig. 5A, B, D, E). However, no effect of TGF-β/EGF treatment was observed at the transcriptional level for CAR or occludin (**Fig. 5G, H**), although E-cadherin mRNA was significantly reduced in cells treated with TGF-β/EGF (**Fig. 5I**),. Expression of the mesenchymal protein vimentin increased at the protein level (**Fig 5E**), and vimentin and α-SMA mRNA levels also increased (**Fig. 5J, K**) in cells treated with TGF-β/EGF for 72h. Additionally, nuclear accumulation of p-Smad3 and Snail1 was detected in 16HBE14o- cells during TGF-β/EGF-induced EMT (**Fig. 5F**). Increased expression of Snail1 mRNA was also observed in 16HBE14o- cells treated with TGF-β/EGF (**Fig. 5L**).

**Figure 6:**
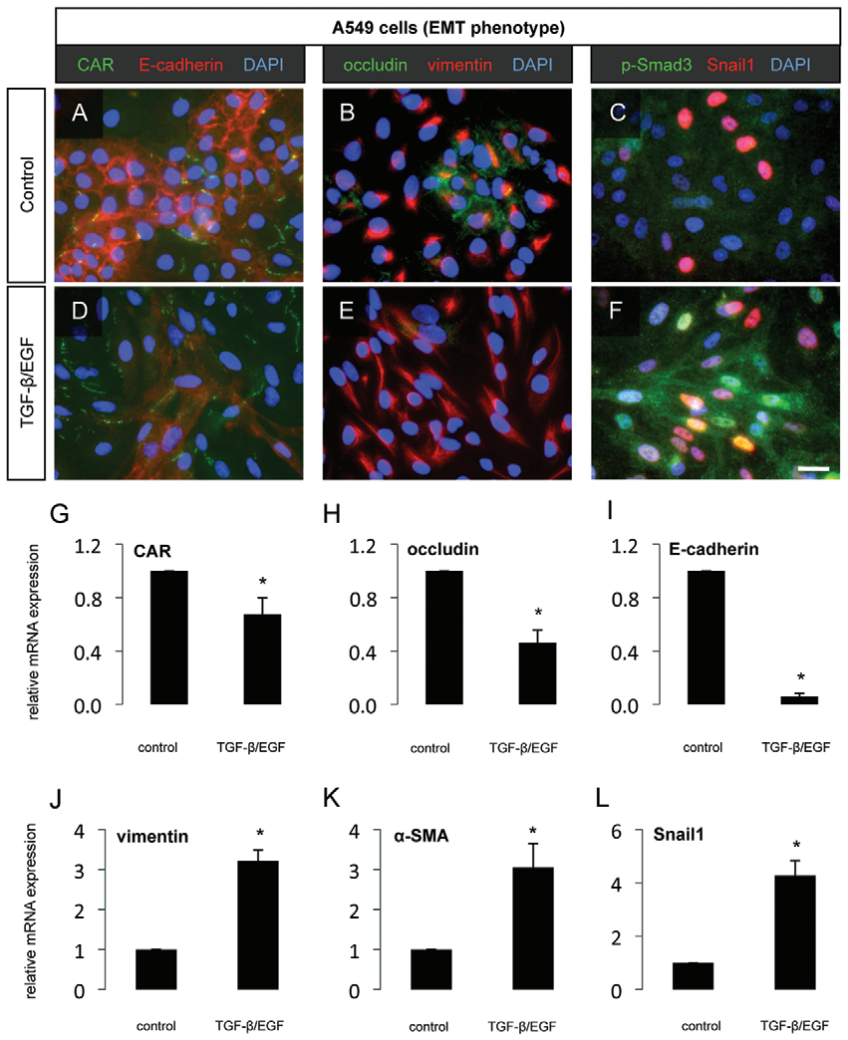
Induction of EMT in A549 cells. Progression through EMT was evaluated in the adenocarcinoma-derived human lung epithelial cell line A549 72h after the addition of TGF-β1 (10 ng/mL) and EGF (50 ng/mL) to the culture medium. The epithelial junction proteins CAR and E-cadherin (A, D), the tight junction protein occludin and the mesenchymal marker vimentin (B, E) as well as the EMT-associated transcription factors pSmad3 and Snail1 (C, F) were assessed by immunofluorescent staining. Scale bar 10 µm. Quantification of the relative mRNA expression of CAR (G), occludin (H), E-cadherin (I), vimentin (J) ), α-SMA (K) and Snail1 (L) was assessed by qPCR.* *p*<0.05 compared to untreated cells. Data represent mean ± SEM, n = 6 from two independent experiments.

Compared to 16HBE14o- cells, A549 airway epithelial cells express significantly lower levels of the epithelial junction proteins CAR, occludin and E-cadherin, which were further decreased following treatment with TGF-β/EGF (**Fig. 6A, B, D, E**). This decrease in epithelial junction proteins was also observed on the mRNA level following TGF-β/EGF (**Fig. 6G, H, I**). A549 cells also exhibited baseline expression of the mesenchymal protein vimentin (**Fig. 6B**), which increased furher following treatment with TGF-β/EGF on the the protein (**Fig. 6E**) and mRNA levels (**Fig. 6J**). Expression of α-SMA mRNA also increased after TGF-β/EGF treatment (**Fig. 6K**). Nuclear accumulation of p-Smad3 and Snail1 was detected in A549 cells at baseline (**Fig. 6C**) and increased during TGF-β/EGF-induced EMT (**Fig. 6F**). Moreover, TGF-β/EGF treatment significantly increased the expression of Snail1 mRNA in A549 cells (**Fig. 6L**).

## Discussion

In this study, we sought to analyze the contribution of EMT to airway remodeling in chronic Th2-polarized inflammation caused by long-term exposure to HDM. We found that airway epithelial cells gradually lost expression of junction proteins and gained expression of mesenchymal proteins, indicative of EMT. Airway epithelial cells undergoing EMT migrated into the subepithelium of larger airways where they were incorporated in accumulating smooth muscle bundles suggesting that EMT contributes to airway remodeling.

The airway epithelium plays a significant role in the pathology of allergic asthma, since these cells serve as a source of multiple cytokines, chemokines, growth factors and other mediators that contribute to the immune response against inhaled allergens [26], [27]. Furthermore, proteolytic components of allergens such as Der p1 have been shown to damage the epithelial barrier by cleaving epithelial tight junction components, thereby enhancing allergen penetration and further exacerbating the immune response [28]. Epithelial damage is also thought to contribute to the development of a dysregulated repair phenotype in the lung, which may lead to airway remodeling in chronic asthma [26], [29].

HDM is a complex mixture of several hundred proteins that represents what human asthmatics are sensitized and exposed to, and is capable of initiating Th2-polarized allergic airway inflammation in the absence of an exogenous adjuvant [30], [31]. Long-term exposure to HDM in mice recapitulates many features of human asthma, including airway hyperresponsiveness, airway wall remodeling and chronic airway inflammation which transitions from a predominantly eosinophilic to neutrophilic inflammatory profile as the disease progresses [23], [31].

Quantification of the number of vimentin+/LacZ+ cells in the airway wall indicated that a significant subset of mesenchymal cells (approximately 30%) was derived from the epithelium. This is in agreement with other studies, in which similar epithelial cell fate-tracking experiments were performed to investigate the contribution of EMT in a mouse model of idiopathic lung fibrosis [32]. Additionally, we observed that, after 10 weeks of HDM exposure, LacZ+ cells in the airway epithelium and subepithelium were capable of producing pro-collagen I, showing that epithelial cells that have progressed through EMT contribute to collagen deposition and subepithelial fibrosis in asthma. Altogether, the data demonstrate that EMT-derived mesenchymal cells significantly contribute to airway wall remodeling in this mouse model of asthma.

TGF-β was significantly upregulated in the lung following allergen challenge, similar to what has been shown in previous studies involving both humans and mice with allergic airway disease [33], [34], [35]. This supports a role for TGF-β signaling in the induction of EMT in HDM-induced airway inflammation. High TGF-β levels in the bronchoalveolar lavage were associated with evidence of EMT in the airway wall. Moreover, a significant overlap between nuclear staining of p-Smad3 and Snail1 was observed, suggesting that Snail1-Smad3/4 repressor complexes could be involved in promoting EMT in airway epithelial cells during HDM induced chronic airway inflammation, similar to what has been shown in breast epithelial cells [12]. Based on these findings, we hypothesize that the mechanism by which EMT occurs during chronic allergic lung inflammation may include elements important to TGF-β-mediated induction of EMT in both cancer and fibrotic diseases.

We next investigated the effect of TGF-β stimulation on cultured airway epithelial cells. In these experiments, TGF-β and EGF co-stimulation induced EMT in human A549 lung carcinoma cells, a well-established model of TGF-β-induced EMT, and, to a lesser extent, in phenotypically normal human bronchial epithelial (16HBE14o-) cells, a phenomenon which has been previously shown in similar studies [19]. Interestingly, we observed that stimulation with TGF-β alone had little effect on the phenotype of 16HBE14o- cells, while this treatment alone resulted in rapid progression to a mesenchymal phenotype in A549 cells (data not shown). However, the addition of EGF to TGF-β treatment in both cell lines resulted in indications of the acquisition of mesenchymal characteristics on both the protein and mRNA levels. Progression into EMT was incomplete in 16HBE14o- cells, with some decrease of protein expression of the tight junction components CAR and occludin, although no change was seen in the mRNA levels of these epithelial markers. Conversely, E-cadherin levels decreased at both the protein and mRNA levels, demonstrating differential regulation of tight junctions and adherens junctions during the initial stages of EMT. These cells also demonstrated increased expression of the mesenchymal markers vimentin and α-SMA, and of activated TGF-β signaling evidenced by nuclear translocation of the EMT-associated transcription factors Snail1 and phospho-Smad3 in treated cells. However, these changes, at least on the protein level, were comparatively mild to what was observed in A549 cells following TGF-β/EGF treatment. These cells demonstrated profound downregulation of all three epithelial junction proteins investigated, both at the protein and mRNA levels, along with a significant upregulation of vimentin and nuclear translocation of Snail1 and phospho-Smad3. Compared to changes in the mRNA expression of mesenchymal proteins, the changes in vimentin, α-SMA and Snail1 mRNA levels were relatively minor in A549 cells stimulated with TGF-β/EGF, likely reflecting the already prominent baseline expression of these markers. Taken together, these results demonstrate that the induction of complete EMT is a gradual, stepwise process, requiring coordinated action of signaling pathways and transcription factors, and regulation of target genes encoding epithelial junction proteins and mesenchymal proteins. These findings lend support to the kinetics of EMT progression *in vivo*, since we observed indications of EMT in the lungs of HDM-exposed mice only at later, more severe stages of the disease (10 weeks of exposure). Further studies are certainly required to investigate the mechanisms of airway wall remodeling at earlier stages of the disease before EMT mechanisms significantly contribute to the population of subepithelial mesenchymal cells in the lung.

In summary, the results of this study provide evidence that EMT contributes to airway remodeling in a mouse model of asthma. Furthermore, they show that EMT during chronic inflammation not only drives phenotypic changes in airway epithelial cells, but also facilitates the migration of these cells to subepithelial regions of the airway wall. Further investigations into the altered migratory capacity of epithelial cells as a consequence of EMT may provide additional mechanistic insight into the role of inflammation in the metastatic spread of tumor cells.

## Supporting Information

Figure S1 Negative control images for immunofluorescent staining. Lung sections from mice exposed to HDM for 15 weeks (A–D) and were stained with the indicated secondary antibodies and imaged under the same conditions as the images in the main body of the study. Isotype control images were stained and imaged under the same conditions as the Snail/pSmad3 images in Fig. 4B–G, but with a rat IgG2a antibody in place of the Snail antibody and an affinity-purified rabbit polyclonal IgG in place of the pSmad3 antibody. Scale bars indicate 10 µm.
(TIF)
