# Supplementary figures and images for: Chronic Respiratory Aeroallergen Exposure in Mice Induces Epithelial-Mesenchymal Transition in the Large Airways
Source: PLoS One. 2011 Jan 20;6(1):e16175. doi: 10.1371/journal.pone.0016175 (PMC3024415; doi:10.1371/journal.pone.0016175)

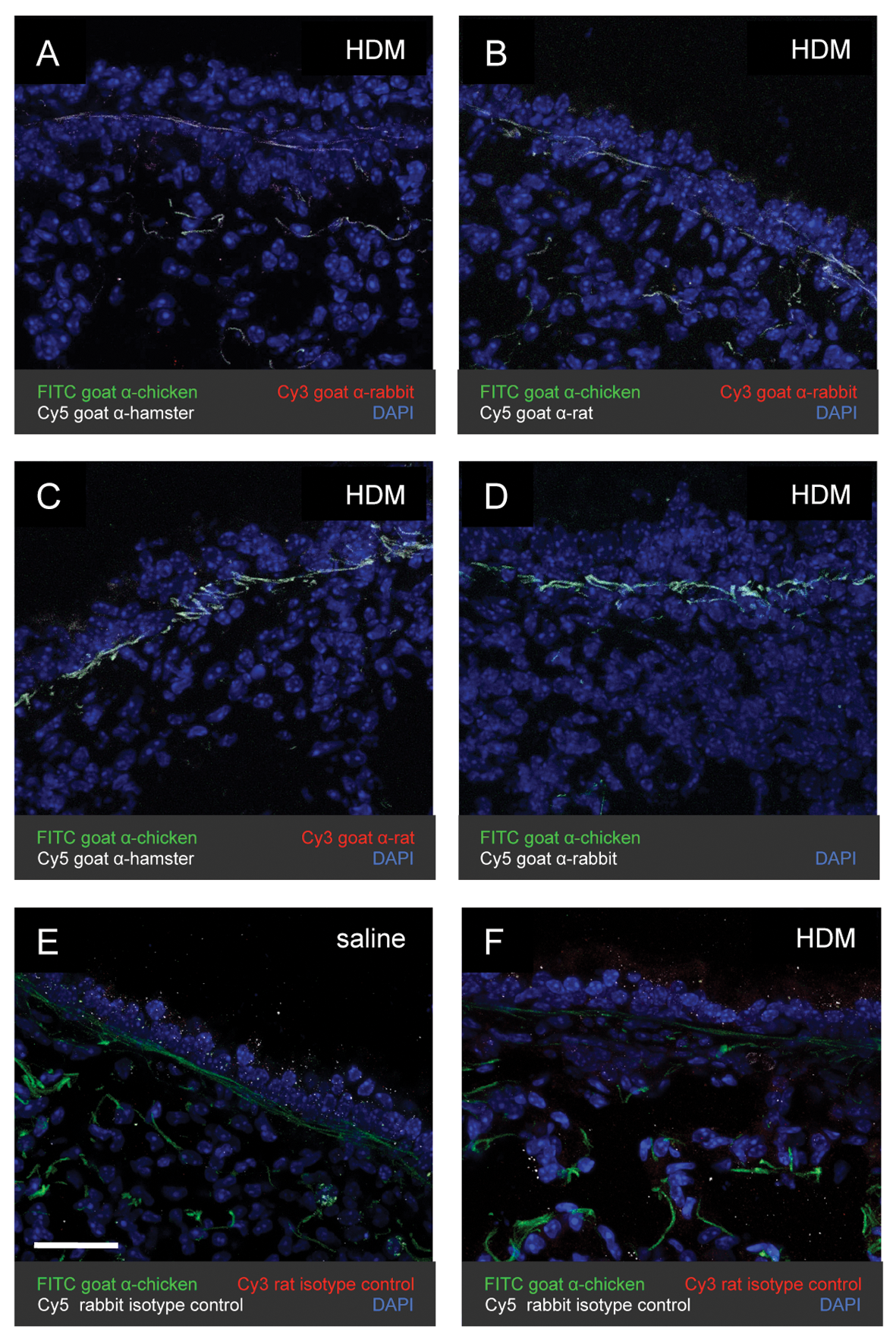

Supplement: Figure S1 — Negative control images for immunofluorescent staining. Lung sections from mice exposed to HDM for 15 weeks (A–D) and were stained with the indicated secondary antibodies and imaged under the same conditions as the images in the main body of the study. Isotype control images were stained and imaged under the same conditions as the Snail/pSmad3 images in Fig. 4B–G, but with a rat IgG2a antibody in place of the Snail antibody and an affinity-purified rabbit polyclonal IgG in place of the pSmad3 antibody. Scale bars indicate 10 µm. (TIF) [file pone.0016175.s001.tif]
